# Prescription Department

**Published:** 1894-03

**Authors:** 


### EPISTAXIS.

Plethoric cases are relieved by:

℞ Tinct. aconit. rad., gtt. viij.
Liq. ammon. acetat., ℥ j.
M. Sig. Teaspoonful every half-hour.

In anemic cases may be employed:

℞ Strychnin. sulphat., gr. $\frac{1}{3}$.

Tr. ferri chlor., ʒ ij.

Vini ergotæ, ℥ ss.

Elixir, ℥ iss.

Aq. destill., q. s. ad ℥ vj.

M. Sig. Tablespoonful three times a day.—*El Siglo Medico.*

### HICCOUGH.

℞. Bismuth, subnitr., gr. xij.
Zinci oxidi,
Zinci valerianat.,
Pulv. calumbæ, ʒ j.
Pulv. opii, gr. iss.
Spirit. anisi, q. s.
M. Sig. Teaspoonful in a glass of sweetened water.—*Ex.*

### A CATHARTIC LEMONADE.

We find the following in one of our English exchanges:

℞ Sodii phosphatis, ʒ vjss.

Spiritus limonis, ♏ xx.

Syrupi simplicis, ℥ ij.

Aquæ destillatæ, ad. ℥ x.

M. Sig. Take at a dose.

### OVARIAN NEURALGIA.

℞ Tr. digitalis, ʒ j.

Tr. gelsemii, ʒ ss.

Potass. bromid., ℥ ss.

Aquæ, ℥ vj.

M. Sig. Tablespoonful every three hours.—*Ex.*

### RHEUMATIC FEVER.

℞ Tinct. aconite root, $\frac{1}{2}$ dr.

Liq. tong. sal., 3 ozs.

Glycerine, 1½ ozs.

Ess. pepsin, 1½ ozs.

M. Sig. Tablespoonful every two hours.

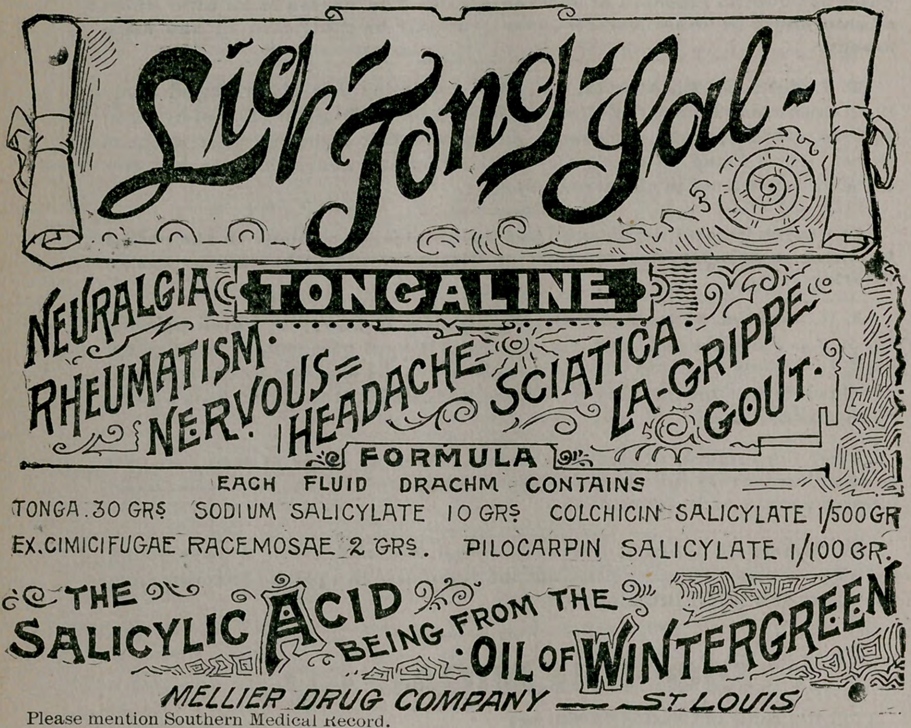

Please mention Southern Medical Record.

CONGESTIVE AND INFLAMMATORY CONDITIONS OF THE EAR.

Dr. Stein commends:

℞ Resorcin gr. iss.

Cocain. hydrochlor., ʒ v–xiiss.

Morphine hydrochlor., gr. ⅙–¾.

Distilled water, ʒ iiss.

M. Sig. For external use by installation.—*Revue de Laryngologie, etc.*

PURGATIVE COMBINATION FOR CHILDREN.

℞ Glycerini, ʒ j.

Ol. cinnamomi, gtt. vj.

Trit. bene et adde

Ol. ricini, ℥ j.

M. Sig. ʒ j. at a dose, pro re nata.—*H. Smith, in Columbus Medical Journal.*

FOR IRRITABLE BLADDER.

℞ Spts. nitre, ʒ ii.
Sanmetto, ℥ ii.
M. Sig. Take a teaspoonful four times a day.

EPILEPSY.

Dr. William Alexander thinks that the best results are obtained from borax, given after the use of bromides, or in connection with small doses of the sodium salt. For some time past he has used the following prescription in a considerable number of cases:—
℞ Sodii biborat., gr. cc.
Sodii bromid., gr. l.
Syr. simpl., ℥ j.
Aquæ, ad. ℥ x.
M. ℥ j ter die in aqua post cidum.
Sometimes, but rarely, a fourth dose has to be given.—*International Medical Magazine.*

LA GRIPPE.

℞ Benzoate sodium, ½ oz.
Glycerine, 1 oz.
Liq. tong. sal., 3 ozs.
Aquæ mentha pip., 2 ozs.
M. Sig. Tablespoonful every two to four hours.

